# Changes in functional connectivity in relapsing-remitting multiple sclerosis spinal cord measured via region-based and data-driven analyses

**DOI:** 10.1162/IMAG.a.51

**Published:** 2025-06-20

**Authors:** Atlee A. Witt, Anna J. E. Combes, Anirban Sengupta, Xinyu Zhang, Seth Stubblefield, Colin D. McKnight, Trey McGonigle, Megan McGrath, Isabella Stewart, Grace Sweeney, Logan E. Prock, Delaney Houston, Simon Vandekar, Kristin P. O’Grady, Baxter Rogers, John Gore, Seth A. Smith

**Affiliations:** Vanderbilt University Institute of Imaging Science, Vanderbilt University Medical Center, Nashville, TN, United States; School of Medicine, Vanderbilt University, Nashville, TN, United States; NMR Research Unit, Queen Square Multiple Sclerosis Centre, UCL Queen Square Institute of Neurology, University College London, London, United Kingdom; Department of Radiology and Radiological Sciences, Vanderbilt University Medical Center, Nashville, TN, United States; Department of Biostatistics, Vanderbilt University, Nashville, TN, United States; Department of Biomedical Engineering, Vanderbilt University, Nashville, TN, United States

**Keywords:** spinal cord, functional magnetic resonance imaging, magnetic resonance imaging, multiple sclerosis, independent component analysis

## Abstract

The clinical picture of persons with multiple sclerosis (pwMS), a neuroinflammatory disease characterized by demyelination, does not consistently correlate with pathology noted on clinical magnetic resonance imaging (MRI). Functional MRI (fMRI) is a valuable tool to understand neural network alterations resulting from structural damage, with studies in the brain employing both region-of-interest and data-driven assessment of functional connectivity. However, similar studies in the spinal cord (SC) remain limited given the challenge of imaging a small structure in an area with substantial physiologic noise. We sought to apply resting-state fMRI at 3T in the SC of healthy controls (HC) and persons with relapsing-remitting MS (pwRRMS) to assess differences in functional connectivity and relate functional markers to clinicial measures of disability. Consistent with prior SC studies, we determined strongest functional connectivity between ventral gray matter horns in both HCs and pwMS and diminished mobility associated with reduced functional connectivity. Using independent-component analysis, we observed a possible compensatory mechanism of increased connectivity in earlier compared with later stages of relapsing-remitting MS. Further exploration is warranted, and our findings support the notion of functional alterations in the SC of pwMS.

## 1 Introduction

Multiple sclerosis (MS) is a debilitating disease of the central nervous system (CNS) that causes demyelination and axonal loss, leading to symptoms such as motor weakness and sensory impairment. MS is one of the most common disabling neurologic diseases impacting young adults, so early diagnosis and effective symptomatic treatment are critical to protect quality of life (Walton et al., 2020). Nevertheless, it is estimated that 20% of persons diagnosed with MS are misdiagnosed (Kaisey et al., 2019). Spinal cord (SC) magnetic resonance imaging (MRI) is recommended in persons undergoing an evaluation for suspected MS and could provide further diagnostic information, especially as the SC is already listed as one of the four CNS regions used to confirm dissemination in space under the 2017 McDonald criteria for diagnosing MS. With almost 9 in 10 persons with MS (pwMS) having SC lesions (Moccia et al., 2019), and cord lesions being specific for demyelinating disease (Trip, 2005), detection of SC lesions can be used to support an MS diagnosis when brain MRI findings are inconclusive (Filippi et al., 2019) and thus lead to increased diagnostic accuracy.

Despite their prevalence in pwMS, the number and volume of lesions do not always correlate with the diversity and severity of clinical outcomes in MS as described by the clinico-radiological paradox (Chard & Trip, 2017). Thus, additional factors may drive clinical deterioration. Beyond just structural changes, widespread functional changes as measured by resting-state functional MRI (rs-fMRI) have been noted in the brains of pwMS (Cader et al., 2006). While task-based fMRI has been applied in the SC of pwMS (Agosta et al., 2008;Rocca et al., 2022;Valsasina et al., 2010,^2012^), applications of rs-fMRI have been more limited (A. Combes et al., 2023;Conrad et al., 2018). Rs-fMRI detects temporally coherent fluctuations in MRI signals that correspond to functionally connected regions without an external stimulus (Hillman, 2014;Kong et al., 2014) and has been shown to be feasible for assessing both healthy and damaged human SC (Barry et al., 2016;Conrad et al., 2018). In a previous study, persons with relapsing-remitting MS (pwRRMS) and healthy controls (HCs) showed greatest functional connectivity (FC) between gray matter (GM) ventral horns, followed next by bilateral dorsal horns and ipsilateral (ventral-dorsal) connectivity, with pwRRMS demonstrating decreased connectivity between ventral horns in the presence of ventral column lesions. Furthermore,Conrad et al. (2018)reported measures of increasing disability are related to increased bilateral connectivity, suggesting a compensatory mechanism among bilateral spinal cord networks as highlighted by rs-fMRI. Similar findings of altered FC, specifically greater FC between ventral horns compared with dorsal horns in both HCs and pwRRMS, have been reported (A. J. E. Combes et al., 2022;Kong et al., 2014). Alterations in FC may directly reflect changes in disability and correlate with neurologic changes in pwRRMS, and thus justify an extensive investigation.

Previous studies of FC in the SC examined connectivity patterns derived from pre-defined regions of interest (ROIs) in the GM (butterfly divided into quadrants, i.e. two ventral and two dorsal horns) and calculated the seed-based FC between the average time series for each GM quadrant (Eippert et al., 2017). However, pre-defined seed- and ROI-based analyses are limited in the ability to detect FC differences in different areas of the GM and may miss different connectivity patterns within an ROI. Data-driven analysis, in contrast, does not require prior information to inform selection of time courses from which to draw comparisons. Independent component analysis (ICA) can be used to infer locations of synchronous BOLD activity in the brain and SC by extracting multiple detectable networks within and among subjects (A. Combes et al., 2023;Kong et al., 2014;Lv et al., 2018;McKeown, 2003;Sengupta et al., 2021) and performing inter-network connectivity analysis. Unlike seed-based approaches, ICA requires no knowledge of the structure of neural networks, as the analysis identifies signals that have a common temporal behavior. ICA is useful to characterize resting-state fluctuations at the individual subject level and can be extended to group-level analyses via temporal concatenation and dual regression (Beckmann et al., 2009). By exploiting temporal fMRI information via ICA, it is possible to identify relevant networks at the group level specific to the population of interest (Beckmann et al., 2009). To our knowledge, this is one of the first studies to apply spatial ICA to the cervical SC of pwRRMS, especially in a cohort of this size.

In this study, we sought to (1) apply rs-fMRI FC to cohesively assess differences in cervical SC functional integrity between HCs and pwRRMS, (2) provide a data-driven analysis of functionally connected regions via ICA, and (3) relate markers of synchronous BOLD activity to clinical measures of disability.

## 2 Methods

### 2.1 Data acquisition

Collections of anatomical and fMRI data were approved by the Vanderbilt Institutional Review Board and performed in accordance with relevant guidelines and regulations. Inclusion criteria for pwMS included relapsing-remitting MS disease status, expanded disability status scale (EDSS) <5, and no contraindications to 3T MRI (Kurtzke, 1983). Studies were performed in 49 HCs and 74 pwRRMS and demographics are provided inTable 1. Participants were also asked to perform sensorimotor testing, including the Timed Up and Go (TUG) (Sebastião et al., 2016), timed 25-foot walk (T25w), and vibration threshold measurements from the right and left great toe using a Vibratron-II device (Newsome et al., 2011). The average of two trials was calculated for the TUG and T25w tests. Participants were scanned on a 3T Philips Elition X (Philips Medical Systems, Best, The Netherlands) using a dual-channel transmit body coil and 16-channel neurovascular coil for signal reception and all images were centered in the cervical SC between C3 and C4 and encompassing C2–C5 cervical vertebral levels. Physiological data, such as respiratory and pulse traces, were acquired using chest bellows and pulse oximeter. Conventional T2-weighted MRI acquisitions covered the same superior-inferior field of view. The acquisition protocol included:

**Table 1.** Demographic and clinical averages with standard deviation for healthy controls (HC) and patients with relapsing-remitting multiple sclerosis (pwRRMS).

	Healthy controls (n = 49)	Multiple sclerosis (n = 74)	*p* -Value
Sex at birth	29F/20M	54F/20M	NS
Age (years)	31.76 +/- 6.72	36.65 +/- 7.55	<0.001**
Disease duration (years)	-	7.90 (0.1 – 20.0)	
EDSS (median score)	-	1 (0-4)	
TUG (s)	6.11 +/- 0.90	7.38 +/- 1.93	<0.001**
T25w (s)	4.22 +/- 0.62	4.87 +/- 1.14	<0.001**
Vibration threshold (left)	1.32 +/- 0.51	2.14 +/- 1.35	<0.001**
Vibration threshold. (right)	1.27 +/- 0.58	2.01 +/- 1.32	<0.001**

*p< 0.05, **p< 0.01.

EDSS = Expanded Disability Status Scale; TUG = Timed Up and Go; T25w = Timed 25-Foot Walk.

- Sagittal T2-weighted turbo spin echo (TR/TE = 2500/100 ms, α = 90°, FOV = 160 × 250 mm^2^, 18 slices, voxel size 0.8 × 1 × 2 mm^3^)
- Axial T2*-weighted multi-slice multi-echo gradient echo (mFFE) (TR/TE/DTE = 700/8/9.2 ms, α = 28°, FOV = 160 × 160 mm^2^, 14 slices, voxel size 0.65 × 0.65 × 5 mm^3^)
- Resting-state functional MRI (3D axial, gradient echo, multi-shot EPI, volume acquisition time = 2.46 s, TE = 20 ms, α = 8°, FOV = 150 × 150 mm^2^, 14 slices, voxel size 1 × 1 × 10 mm^3^(reconstructed 0.59 × 0.59 × 5 mm^3^) 200 dynamics (∼8 min), EPI factor = 9).

### 2.2 Anatomical image processing

The image processing steps were adapted from prior work and used the Spinal Cord Toolbox (SCT, version 6.0) and FSL (Barry et al., 2016;A. J. E. Combes et al., 2022;De Leener et al., 2017;Jenkinson et al., 2012;Smith et al., 2004). Cord segmentations were obtained on sagittal T2 and axial mFFE images prior to identification of vertebral levels on sagittal T2, co-registration of the T2 and mFFE images, and creation of GM and white matter (WM) masks on the mFFE image. Unsatisfactory segmentations were corrected manually. Lesions were identified and masks manually drawn by an experienced neuroradiologist using ITK-Snap (Yushkevich et al., 2006). Total cord volume and lesion volume were calculated using*fslstats*constrained between vertebral levels C3, C4, and C5*,*and cross-sectional area (CSA) was calculated within C3 using SCT’s*sct_process_segmentation*. Lesion burden was calculated as the total lesion volume divided by the total cord volume within the specified vertebral levels [C3, C4, C5, C3–C5].

### 2.3 Functional image processing

**Fig. 1.**
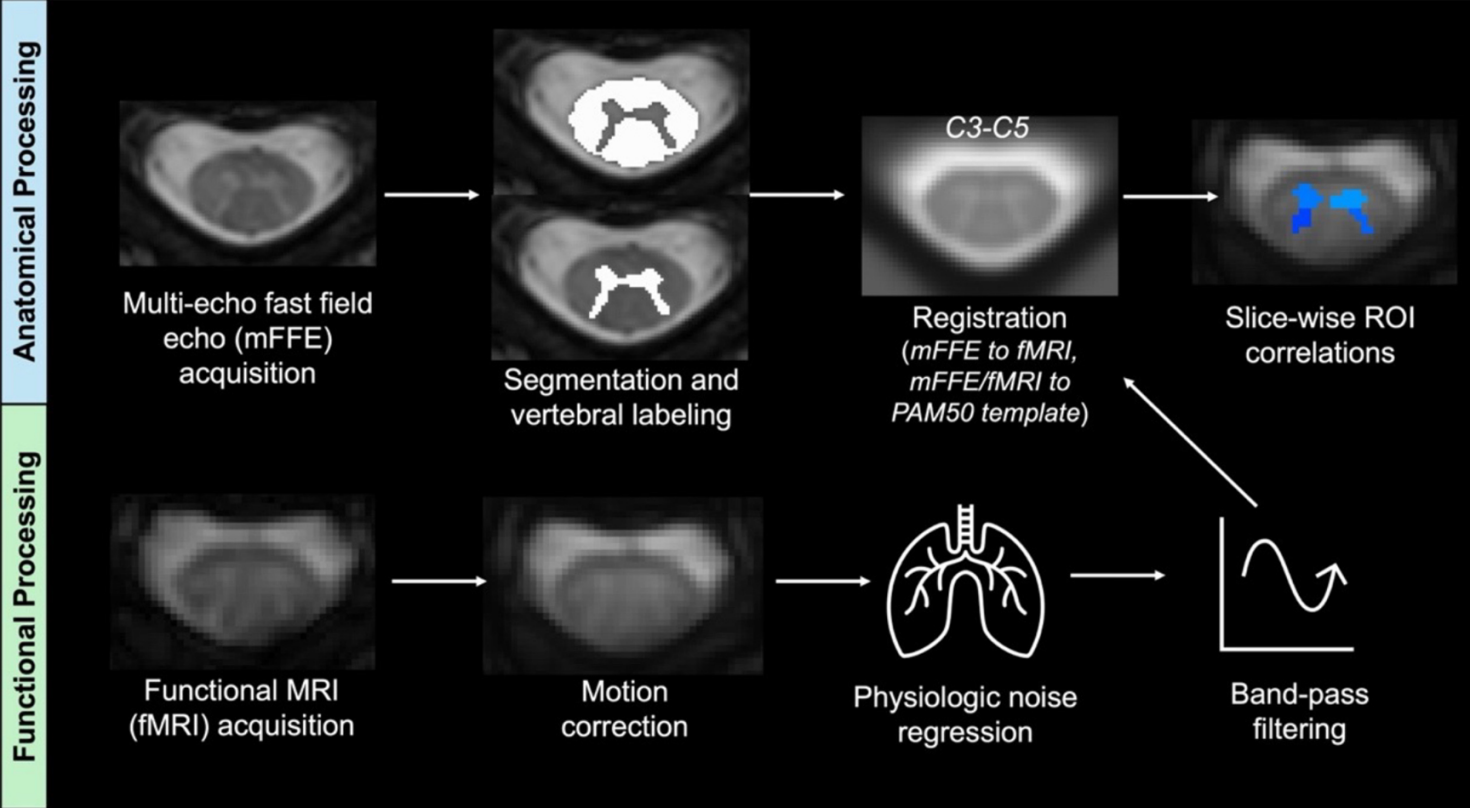
Anatomic and functional data processing pipelines. For the anatomic image, vertebral levels were identified on the sagittal T2w image before co-registration of the T2w and multi-echo fast field echo (mFFE) image. For the functional image (fMRI), motion correction was followed by physiologic noise regression using AFNI-RETROICOR and band-pass filtering via a Chebyshev Type II filter. The resulting denoised fMRI and mFFE images were co-registered to one another, and then to the PAM50 template between spinal levels C3 and C5. The gray matter (GM) horns applied on top of the final functional image were extracted from the mFFE image in functional space. ROI correlations were identified between each horn, per slice.

Resting-state functional data were preprocessed using SCT, Matlab R2023b, and Python 3.12.0. Motion correction was performed on the fMR images using SCT’s*sct_fmri_moco*function. CSF and cord masks were produced via in-house code on the motion-corrected images before denoising of physiological signals related to respiratory and cardiac motion via AFNI-RETROICOR (Glover et al., 2000). Noise related to physiologic data, CSF signal, background anatomical signal outside of the spine, and motion correction parameters were regressed out for each voxel using principal component analysis (Barry et al., 2018;Conrad et al., 2018). The resulting denoised time series from the functional data was band-pass filtered via a Chebyshev Type II filter with cutoffs of 0.01 and 0.10 Hz. Before further analyses, the mFFE and functional images were co-registered to each other and to the PAM50 template cropped between C3 and C5 (De Leener et al., 2018) (Fig. 1).

### 2.4 ROI-based analysis

To calculate correlations between the pre-processed time series, ROIs were extracted for each GM horn (left ventral [LV], right ventral [RV], left dorsal [LD], right dorsal [RD]) on the GM-mask derived from the mFFE co-registered to functional space. Then, the time -series from each horn were correlated with each other and the Pearson correlation coefficients were reported slice wise for each of six pairs of regions: ventral-ventral, dorsal-dorsal, ipsilateral ventral-dorsal left, ipsilateral ventral-dorsal right, contralateral right-ventral left-dorsal (CRVLD), and contralateral left-ventral right-dorsal (CLVRD). For each subject, the slice-wise correlation relating to each region was averaged to produce a single value for each of C3, C4, C5, or C3 through C5 spinal levels. The slice-wise temporal signal-to-noise ratio (tSNR) was calculated as the mean signal over time divided by the standard deviation following denoising via RETROICOR. The tSNR was calculated for data encompassed by either C3, C4, C5, or C3 through C5.

To perform consistently robust statistical analysis on the ROI-based connectivity, pwRRMS subject data were removed if no EDSS score was recorded (5), no sensorimotor testing was recorded (2), motion in mFFE image was excessive as judged by trained neuroradiologists C.M. and S.S. (2), and no lesion masks were drawn (4). Image data from 8 HCs and 14 pwRRMS were excluded due to poor overall fMRI or anatomical image quality as judged by 2 independent raters [A.W. and M.M.] leaving 41 HCs and 47 pwRRMS for final ROI analysis.

### 2.5 Statistical analysis for region-based FC

R (version 4.4.0) was used for all statistical analyses (R Core Team, 2024). Demographic and clinical information were compared between HCs and pwRRMS using Welch*t*-tests for continuous variables and a chi-squared test for sex at birth. Two-sample independent t-tests were used to assess significant differences between HCs and pwRRMS for each ROI, followed by paired t-test to assess significant differences between ROIs for each cohort [e.g. ventral-ventral vs. dorsal-dorsal within HCs, ventral-ventral vs. dorsal-dorsal within pwRRMS]. A Benjamini–Hochberg*p*-value correction was applied to control the false discovery rate across tests separately for the two-sample and paired tests.

Our investigations into ROI-based FC and its relationship with sensorimotor tests were grouped into measures of mobility, vibration threshold sensitivity, and clinical measures of disability such as EDSS. We conducted three models with FC as the outcome variable (six region pairs) and primary covariates of motor function (TUG and T25w), vibration sensation, and EDSS. For mobility measures, we computed an aggregate mobility time index by Z-scoring TUG and T25w variables across all participants and averaging the Z-scores for each participant (Z-scored TUG/T25w). The vibration sensation measures were also Z-scored and averaged across the left and right sides. For both mobility and vibration models, additional covariates included age, sex at birth, SC segment (C3, C4, C5), and diagnosis (HC, MS) as well as random effects for participant. We tested the interaction between diagnosis and the mobility/vibration threshold index, followed by testing the main effect.

The third linear mixed effect model was fit in the MS patients only to determine if FC was related to EDSS, with covariates including age, sex at birth, lesion burden, disease duration, and SC segment (C3, C4, C5). EDSS was treated as a continuous variable. For all analyses, tests were performed using “type 2” sum of squares, so that main effects are tested without interactions in the model. We used robust standard errors and Benjamini–Hochberg*p*-value correction to account for multiple tests across the FC outcomes. Due to the exploratory nature of this study, we report both adjusted and unadjusted*p*-values for the three models investigating FC with mobility, vibration, and EDSS.

### 2.6 Independent-component analysis (ICA)

To perform ICA analysis, the rs-fMRI data were co-registered to the PAM50 template using*sct_register_multimodal*(step 1: segmentation-based, affine; step 2: image-based, symmetric normalization) with*sct_resample*for interpolation of the template image (0.1 × 0.1 × 0.2 mm^3^, 198 slices). The resulting functional images in PAM50 space were cropped between vertebral levels C3–C5 and entered into the Group ICA of fMRI Toolbox (GIFT) software in Matlab with a reference GM mask cropped to the same levels (Calhoun et al., 2001,^2005^,^2009^). The 4D functional data were concatenated to a 2D matrix consisting of spatial and temporal information and ICA was performed to estimate independent spatial components and a matrix of corresponding temporal dynamics. We explored analyses with 10, 20, 30, 40, and 50 components for 10 pwMS and 10 HCs to select the appropriate component number. Based on these results and existing literature (Sengupta et al., 2021), we selected a total of 30 components to best represent component signals from the C3–C5 segments, which showed greatest agreement with the GM parcellation of the SC from the SCT, and to maintain statistical power without overestimating independent components. We considered vertebral levels to be C3, C4, and C5, with components located between vertebral levels (i.e. between vertebral level C3 and C4) as C3–C4 or C4–C5.

We used the dual-regression analysis included with the GIFT^22^toolbox to obtain subject-specific component maps. This involved using group-level spatial maps as spatial regressors to find associated temporal dynamics, followed by identification of subject-specific maps using the associated temporal dynamics as temporal regressors (Beckmann et al., 2009). To visualize the spatial components overlaid on the PAM50 atlas in FSLeyes, z-scores were thresholded between 5 and 15. Three independent raters (C.C., S.S., A.W.) determined the anatomical location of each of the 30 components: LV, left mid (LM, between left ventral and dorsal horns), LD, central commissure (C), RV, right mid (RM, between right ventral and dorsal horns), and RD. To ensure adequate data quality, the full cohort of 74 pwRRMS was analyzed, and participants with missing EDSS scores (5) followed by a tSNR below the 25^th^percentile of the mean tSNR for pwRRMS were removed from further analysis. Exclusion criteria was determined separately for ICA and ROI-based analyses, respectively, given the ROI analysis encompassed greater signal across more voxels and thus tSNR exclusion may be less applicable.

For the remaining participants, the between-network connectivity was calculated as the correlation (Pearson’s R-values) between time courses of pairs of components and then averaged across participants to produce connectivity matrices. We calculated the difference in average FC between HC and MS subgroups, including pwRRMS with an EDSS of 0, 1, and more than 2 [EDSS 2 +], and thresholded values between -0.12 and 0.08 for visualization. The group distributions included N = 37 HCs, N = 16 EDSS = 0, N = 20 EDSS = 1, N = 14 EDSS = 2+ (accounting for the pwRRMS with no recorded EDSS score).

For the difference between correlation matrices (i.e. subtraction between group-average HC connectivity and pwRRMS connectivity), each EDSS group was compared with the HC group using a Welch’s t-test.

## 3 Results

### 3.1 Participant demographics

Demographic and clinical variables for all participants, except for sex at birth, were significantly different between HCs and pwRRMS with pwRRMS having longer TUG and T25w times and higher vibration thresholds for both the left and right toe. PwRRMS were also significantly older than the HC counterparts (Table 1).

### 3.2 ROI-based FC and group measures

**Fig. 2.**
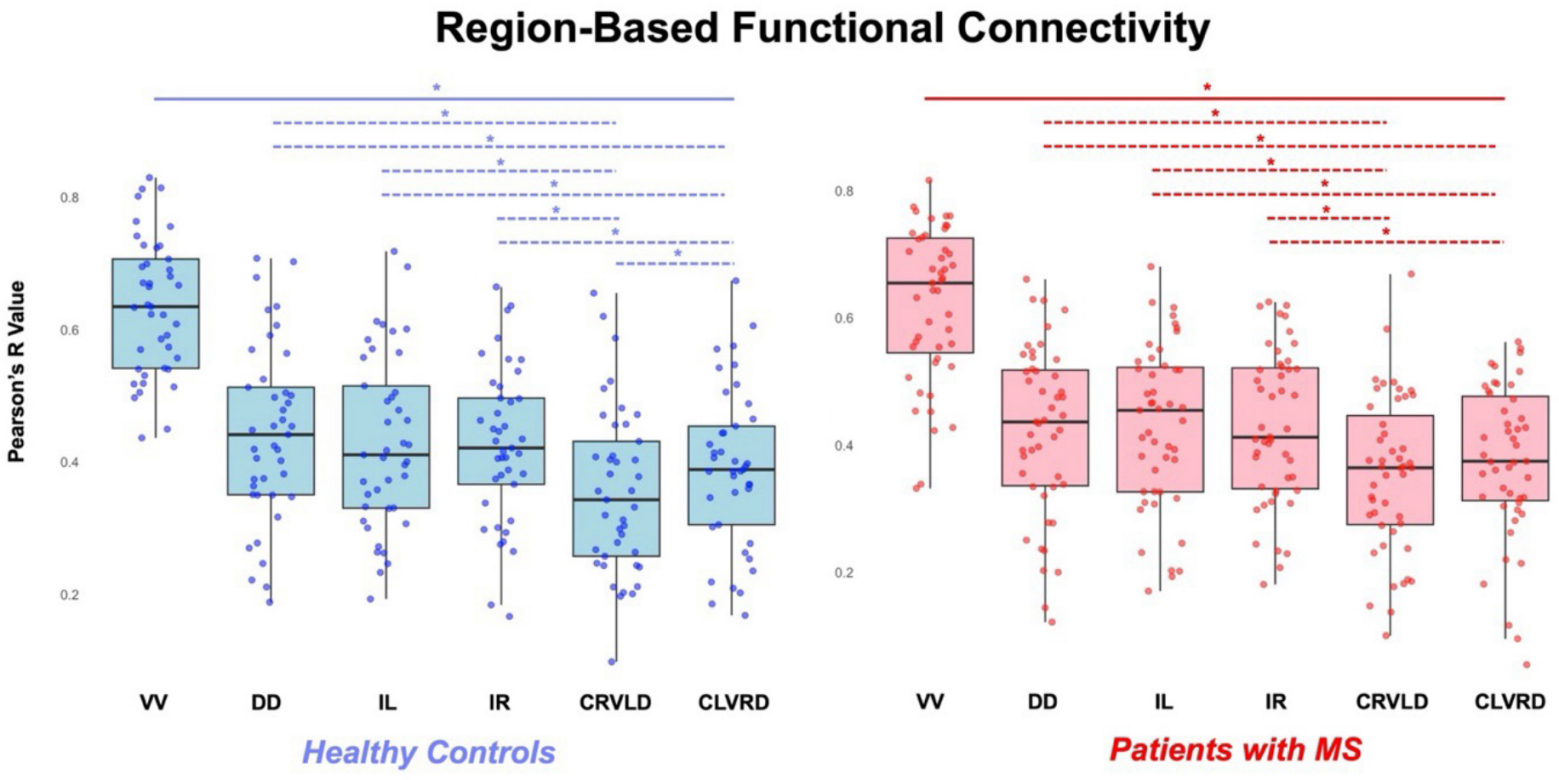
Connectivity between ROIs, compared between HCs and pwRRMS. Significant differences in connectivity are listed in blue for differences between HC regions and red for differences between pwRRMS regions. The solid lines represent significant differences between VV connectivity and the remaining five regions, while the dotted lines represent significant differences between individual regions. *p<0.05

For ROI-based FC, average Pearson’s R values and tSNR for each GM sub-region are listed inTable 2. There were no significant differences in tSNR, averaged across all 14 slices per subject, for both HCs and pwRRMS when split into C3–C5, C3, C4, and C5 vertebral levels. FC was assessed at the group level between C3 and C5 vertebral levels. There were no significant differences between HC and pwRRMS values for any of the six regions. When looking at the adjusted*p*-values for each paired t-test, ventral-ventral FC was significantly higher than FC for all other regions [dorsal-dorsal, ipsilateral left, ipsilateral right, CRVLD, CLVRD] for both HCs and pwRRMS (*p<*0.010). For HCs and pwRRMS, dorsal-dorsal, ipsilateral left, and ipsilateral right were significantly greater than both CRVLD and CLVRD (*p<*0.010) (Fig. 2). CRVLD had the lowest connectivity for both HCs and pwRRMS compared with the five other regions.

**Table 2.** Average connectivity, represented by Pearson correlation coefficients, between regions of interest (ROIs).

	VV (mean)	DD (mean)	IL (mean)	IR (mean)	CRVLD (mean)	CLVRD (mean)	tSNR (mean)
HC							
C3–C5	0.64 +/- 0.10	0.44 +/- 0.13	0.43 +/- 0.13	0.43 +/- 0.12	0.35 +/- 0.12	0.39 +/- 0.12	21.58 +/- 4.32
C3	0.62 +/- 0.12	0.45 +/- 0.15	0.41 +/- 0.15	0.40 +/- 0.15	0.34 +/- 0.14	0.39 +/- 0.14	23.84 +/- 4.70
C4	0.66 +/- 0.13	0.45 +/- 0.13	0.43 +/- 0.14	0.44 +/- 0.13	0.36 +/- 0.13	0.39 +/- 0.14	21.10 +/- 4.21
C5	0.63 +/- 0.14	0.43 +/- 0.17	0.46 +/- 0.17	0.43 +/- 0.17	0.35 +/- 0.18	0.39 +/- 0.17	19.55 +/- 4.84
pwRRMS							
C3–C5	0.62 +/- 0.12	0.42 +/- 0.13	0.43 +/- 0.13	0.43 +/- 0.13	0.35 +/- 0.12	0.38 +/- 0.12	20.12 +/- 5.18
C3	0.61 +/- 0.12	0.43 +/- 0.17	0.41 +/- 0.16	0.41 +/- 0.15	0.34 +/- 0.17	0.37 +/- 0.16	22.70 +/- 5.55
C4	0.63 +/- 0.15	0.41 +/- 0.14	0.42 +/- 0.15	0.43 +/- 0.15	0.35 +/- 0.15	0.38 +/- 0.15	19.91 +/- 5.58
C5	0.64 +/- 0.15	0.43 +/- 0.16	0.47 +/- 0.16	0.45 +/- 0.16	0.39 +/- 0.15	0.39 +/- 0.16	17.62 +/- 5.34

Connectivity values are split between C3, C4, C5, and between C3 and C5 vertebral levels.

TSNR (temporal signal-to-noise ratio) values are listed as an average for the same vertebral levels.

VV = ventral-ventral, DD = dorsal-dorsal, IL = ipsilateral left, IR = ipsilateral right, CRVLD = contralateral right ventral left dorsal, and CLVRD = contralateral left ventral right dorsal.

### 3.3 Relationships between ROI FC and sensorimotor variables

We used mixed effect models applied to all Fisher-transformed ROI-based FC metrics to study the association of FC and sensorimotor measures. The association between the mobility index and FC did not significantly differ between HC and MS in any region pair; however, there was a main effect of mobility on FC in dorsal-dorsal, ipsilateral left, and CRVLD regions (*p=*0.004*, p=*0.001*, p<*0.001). The main effect was characterized by a continuous decline in FC with mobility followed by a slight rebound in the MS group due to the smaller sample size. There were no significant associations with FC metrics and vibration when averaged between left and right sides, with evident effect cancelation from both positive and negative trends across the range of vibration measurements. Coefficients from the linear mixed effect model revealed higher dorsal-dorsal, ipsilateral left, CRVLD, and CLVRD FC in females for the mobility model (adjusted*p=*0.015*, p=*0.015*, p=*0.028*, p=*0.015), and higher dorsal-dorsal, ipsilateral left, and CLVRD FC for females in the vibration model (adjusted*p=*0.080*, p=*0.080*, p=*0.080).

**Fig. 3.**
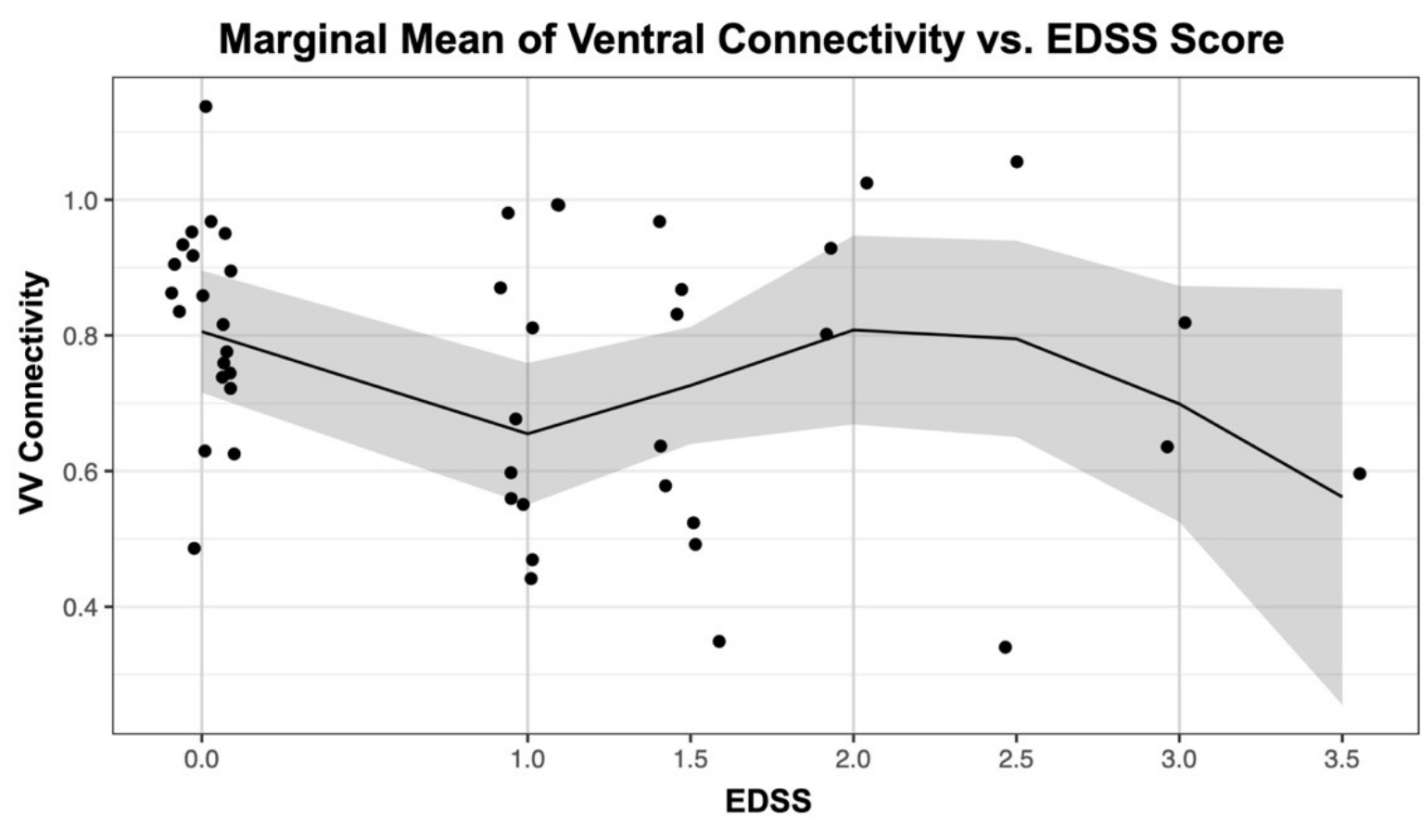
Marginal mean of Fisher-transformed VV connectivity versus averaged EDSS across segments C3, C4, and C5 in pwRRMS. In this case, the marginal mean represents the estimated average FC (i.e. VV) for different subgroups of EDSS, with EDSS 0 as the MS group with no reported clinical disability. The shaded region indicates 95% confidence intervals.

There was a significant nonlinear relationship between ventral-ventral and EDSS, with an unadjusted*p*-value of 0.015 (adjusted*p=*0.093), and a significant negative association from EDSS = 0 to EDSS = 1 with an unadjusted*p*-value of 0.026 (adjusted*p=*0.155;Fig. 3). Given lesion burden was included as a covariate for the EDSS analysis, average lesion burden between C3 and C5 as split according to EDSS group was 0.09 (EDSS = 0), 0.22 (EDSS = 1), 0.11 (EDSS = 1.5), 0.08 (EDSS ≥ 2).

### 3.4 ICA-derived spatial components

**Fig. 4.**
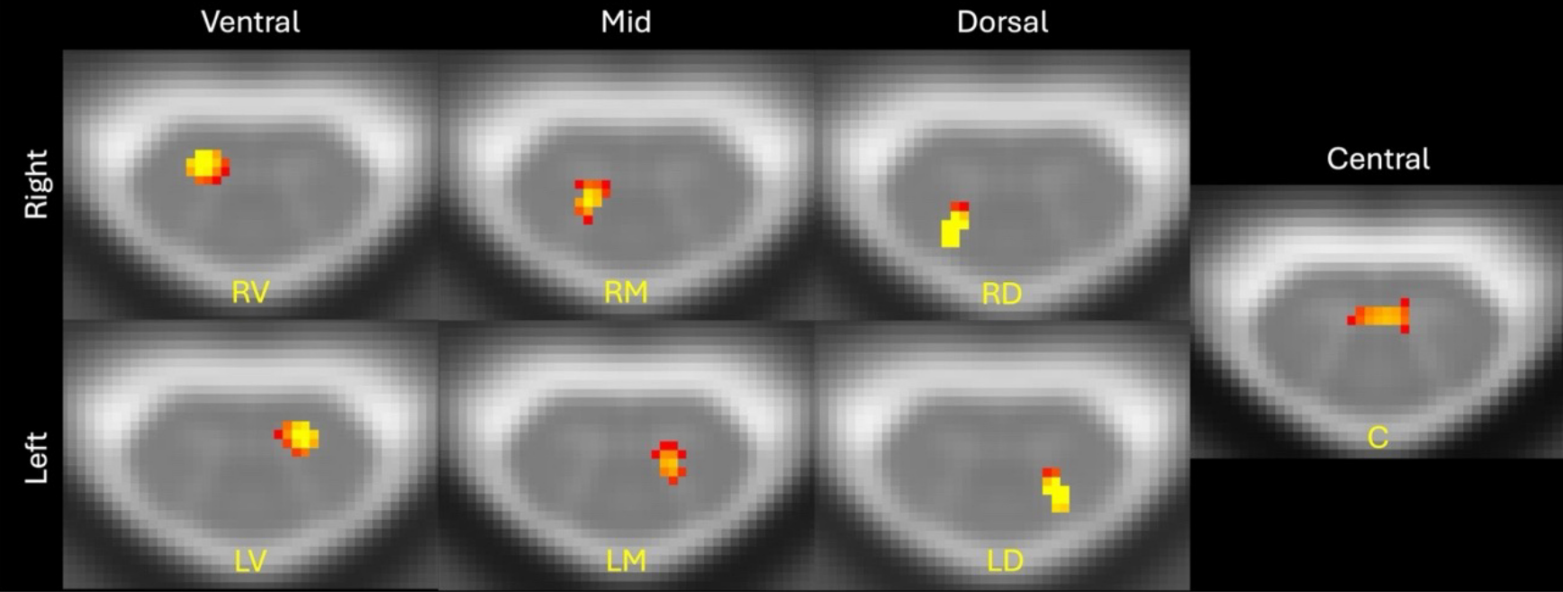
Independent component analysis (ICA) aggregate components, with 7 of the 30 components represented, to demonstrate anatomic specificity of signal localized to the left ventral (LV), left mid (LM), left dorsal (LD), right ventral (RV), right mid (RM), right dorsal (RD), and central (C) separate cord regions. To view the spatial components overlaid on the PAM50 atlas, z-scores were thresholded between 5 and 15.

**Fig. 5.**
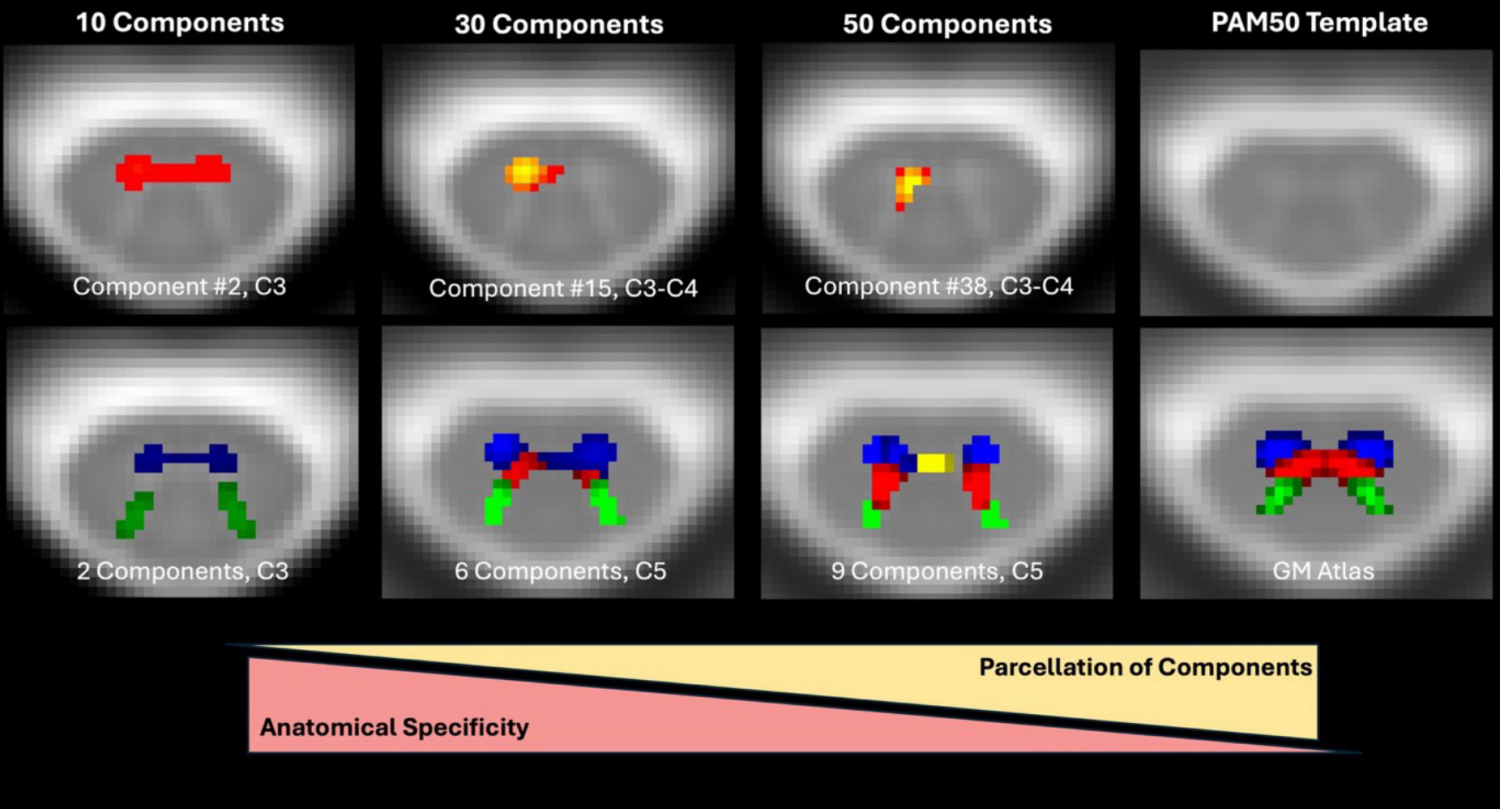
ICA performed with 10, 30, and 50 components. A single component for each analysis is shown in the first row, with overlapping components to produce a “full” GM map shown in the second row with blue indicating ventral components, red for the middle components, green for the dorsal components, and yellow for the central component. The GM mask derived from the PAM50 template is provided for a comparison of standard ventral, middle, and dorsal GM regions. While 50 components demonstrate greatest parcellation, we noted more appropriate anatomic specificity with 30 components (central commissure component not shown at C5).

Following ICA analysis using 30 components, 7 anatomical components were identified by the 3 independent raters on the aggregate ICA data: LV, LM, LD, C, RV, RM, and RD. Though these components were not always conserved across all vertebral levels, each vertebral level [C3, C4, C5] had a component corresponding to at least one of the seven distinct anatomical regions (Fig. 4). For the same analysis performed with 10, 20, 30, 40, and 50 components for 10 HCs and 10 pwRRMS participants, increased number and thus parcellation of components led to decreased anatomic specificity (segregation of GM horns) (Fig. 5). There was correspondence of the six components listed at C5 and the GM atlas from the PAM50 template. Hence, the selection of 30 components provided the most consistent GM functional parcellation in agreement with the GM atlas. While previous studies have not identified components between vertebral levels, our inclusion of between-level components (i.e. C3–C4) mirrors the neuroanatomy of the SC with long-range interneurons, for example (Kong et al., 2014).

### 3.5 Presence of ICA-derived intra-level and inter-level FC

**Fig. 6.**
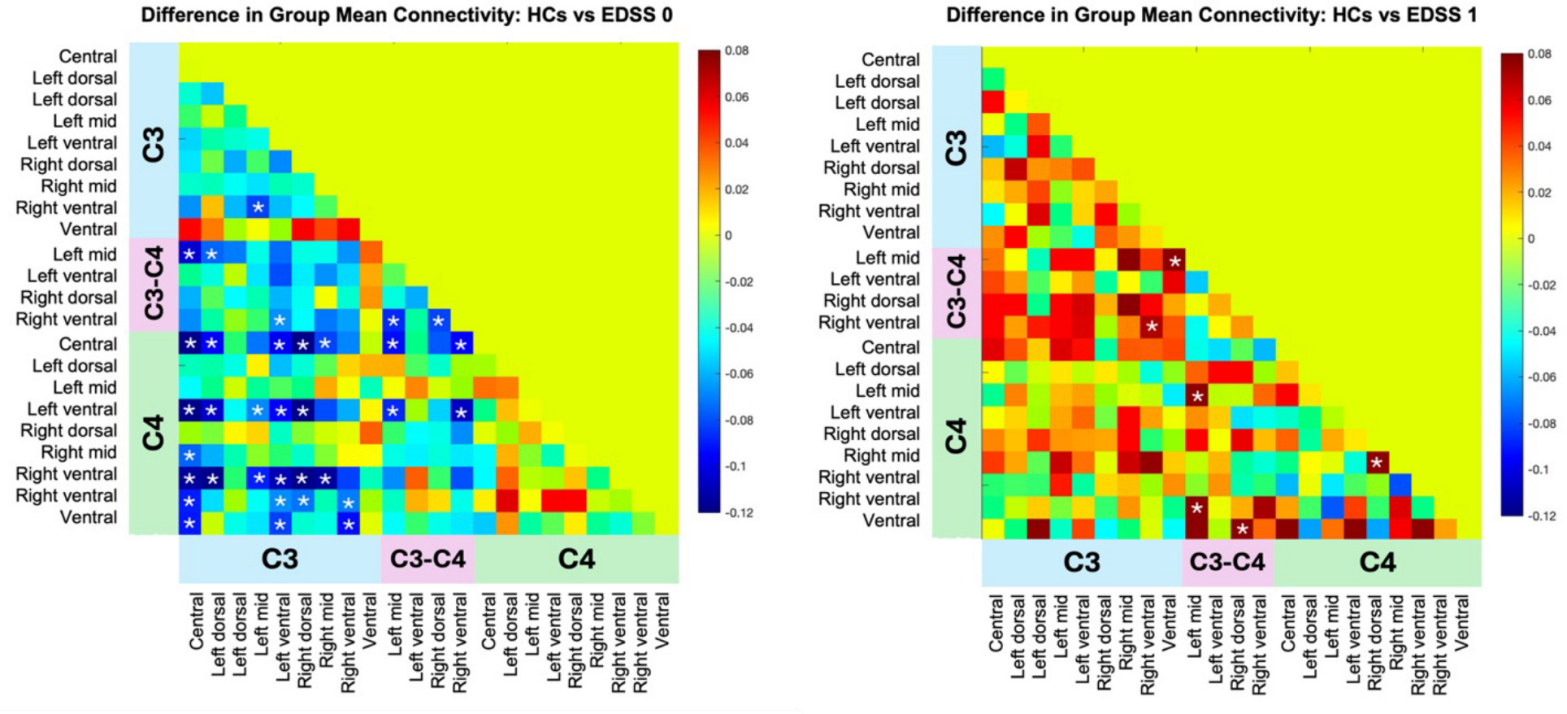
The difference between the average group connectivity values, calculated as the correlation between time courses of pairs of components, between HCs and pwRRMS, with the EDSS 0 cohort on the left and EDSS 1 cohort on the right. The blue indicates greater connectivity in pwRRMS compared with HCs, while the red indicates greater connectivity in HCs compared with pwRRMS. The significant differences (*p<*0.05) calculated via a non-parametric t-test are marked with a “*”.

To minimize edge effects based on the spinal regions imaged [C2–C5], we restricted our ICA on the C3, C3–C4, and C4 levels, reducing the number of visualized components from 30 to 22. The difference between the group-average HC correlation matrix and pwRRMS (EDSS = 0) is highlighted (Fig. 6, left). Out of the 231 correlations, 34 differed significantly between groups (*p<*0.050, indicated by a “*” on the correlation matrix) with all significant correlations indicating higher connectivity in pwRRMS compared with HCs. For EDSS = 0, these 34 correlations included 3 significant intra-level correlations (LM-RV C3, LM-RV and RD-RV C3–C4). The remainder were inter-level, between C3/C4, C3/C3–C4, or C3–C4/C4. There was no evidence of diminished connectivity in pwRRMS compared with HCs between C3 and C4 vertebral levels for pwRRMS with an EDSS = 0.

The same analysis was performed to compare HCs and pwRRMS with an EDSS of 1 and an EDSS of 2 or greater, respectively. The correlation matrix related to pwRRMS with an EDSS = 1 demonstrated six significant differences between groups, with all indicating lower connectivity in pwRRMS compared with HCs (*p<*0.050) (Fig. 6, right). One of these correlations was intra-level (RD-RM C4). The correlation matrix related to pwRRMS with an EDSS = 2+ showed five significant correlations with three of these correlations indicating lower connectivity in pwRRMS compared with HCs (*p<*0.050). One correlation was intra-level at C4 (RM-RV).

An alternative view of these connections, overlaid on the SC vertebral levels, is highlighted inFigure 7. When considering laterality for pwRRMS at an EDSS = 0 compared with HCs between C3 and C4, there are six connections stemming from the right hemicord of C3, four from the central commissure region of C3, and eight from the left hemicord of C3. Additionally, components segregated to the right, central, and left regions of C3 did not demonstrate significant connections solely on that respective side, but rather spanned across all regions (i.e. LV C3 to LV C4, C C4, RV C4). It should be noted that a ventral component of C4 did not demonstrate specific laterality (i.e. left ventral or right ventral) yet still demonstrated strong significant correlations with components in C3. Similarly, though we did not note significant connectivity differences between HCs and pwRRMS with an EDSS = 1 between C3 and C4, we do visualize decreased connectivity in pwRRMS compared with HCs when expanding the analysis to include C3–C4 between-level components.

**Fig. 7.**
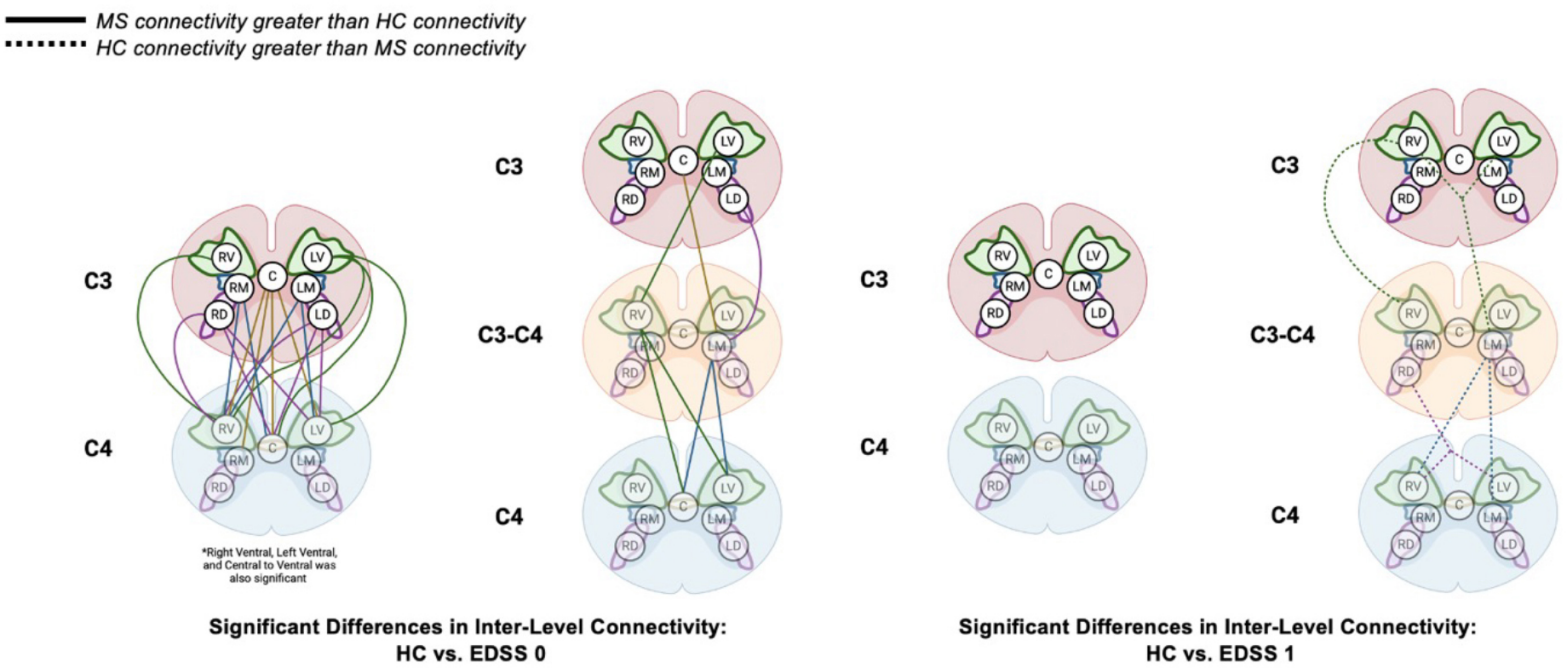
Representation of the significant correlations from the connectivity matrices fromFigure 6overlaid on the vertebral levels. The solid line indicates greater connectivity in pwRRMS compared with HCs and the dotted line indicates greater connectivity in HCs compared with pwRRMS. The between-level components are included in the C3–C4 levels.

When comparing correlations between components, such as component 17 representing an LV spatial component at C4 and component 1 representing a C spatial component at C3, it was noted that not all correlations that are significant when comparing HCs with an EDSS group will be consistently significant across all EDSS groups. Pairs of components compared between HC and pwRRMS split into disease groups (EDSS = 0, EDSS = 1, or EDSS = 2+) are shown inFigure 8, highlighting greater inter-level connectivity in pwRRMS with no clinically reported disability (EDSS = 0,*p*< 0.001) and diminished inter-level connectivity in pwRRMS in the low-disability groups (EDSS = 1, EDSS = 2+) when compared to HCs. For correlations between intra-level components, such as component 19 representing an RM spatial component at C4 and component 18 representing an RD spatial component at C4, pwRRMS in the EDSS = 1 group demonstrated significantly diminished connectivity compared with HCs (*p=*0.015).

**Fig. 8.**
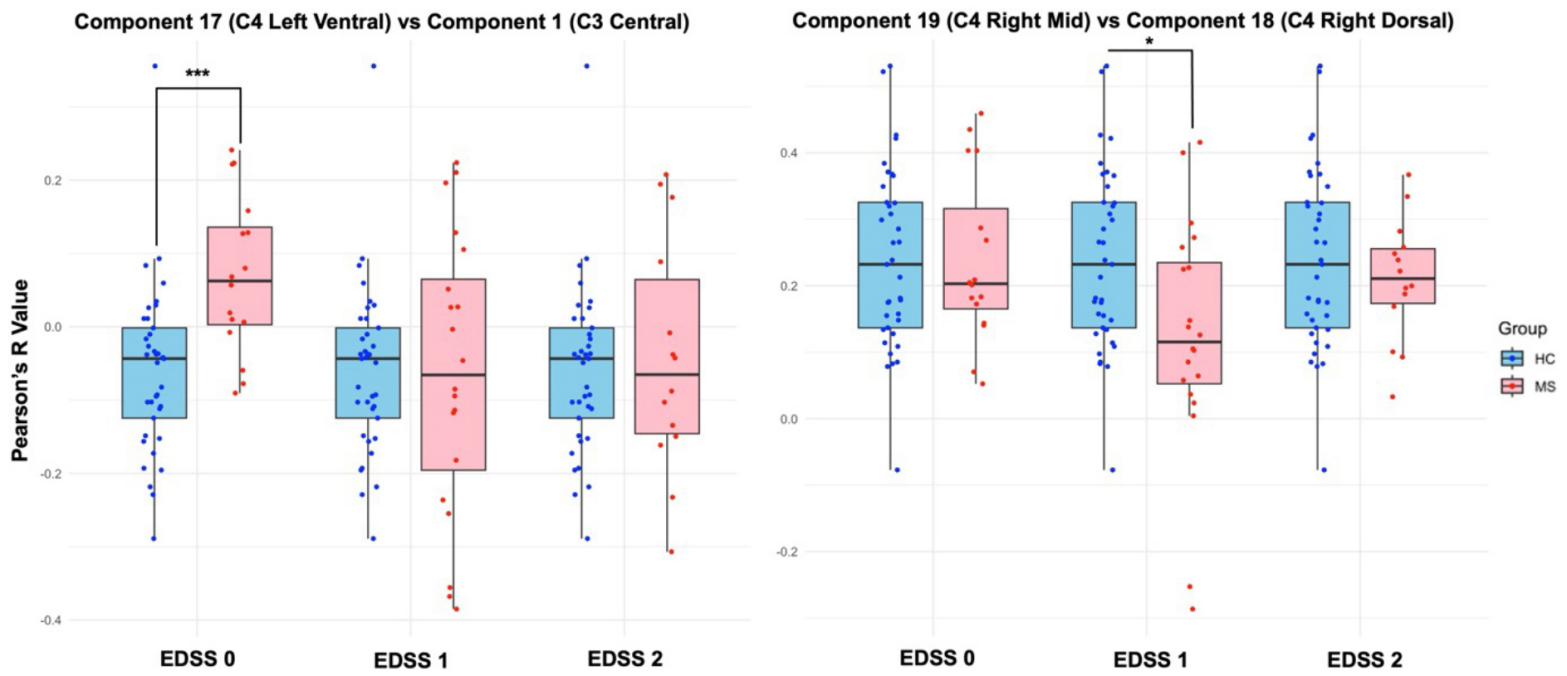
Comparison of the connectivity, or the Pearson correlation coefficients between time courses, between pairs of components for HCs and pwRRMS split into EDSS 0, EDSS 1, and EDSS 2+. Data from healthy controls are repeated across bars. All component pairs were considered, and no component pair demonstrated significant differences across all three disability groups (HC vs. EDSS 0, EDSS 1, EDSS 2+). The selected component pairs highlight diverse correlations across disability groups. *p<0.05,***p <0.001

## 4 Discussion

We hypothesized that resting-state FC relates to sensorimotor and disability measures and may provide a better understanding of the functional health of the SC in individuals with MS, beyond anatomical MRI measures of lesion burden or clinical measures alone. These findings could be used to study the nature of SC functional integrity in MS and evaluate which SC networks are most related to clinical presentation (and potentially progression) of MS, in the same manner that fMRI has been utilized to predict surgical responders and non-responders in refractory temporal lobe epilepsy (Englot et al., 2015;Garner et al., 2019;Kesavadas & Thomas, 2008;Sainburg et al., 2022).

We set out to evaluate both ROI- and ICA-based fMRI analyses in the GM of the SC in HCs and pwRRMS to understand the connectivity patterns distinct to MS patients and identify differences that may exist depending on severity of disability. We found evidence of ROI-based FC patterns correlated with measured mobility, and demonstrated that ICA-derived FC is increased in MS patients with EDSS = 0 and decreased in EDSS > 0 compared with HCs. We also demonstrated that ICA-derived FC can be obtained in the SC at 3T and used to relate functional imaging measures to disability.

### 4.1 Correlations between ROI-based FC and sensorimotor variables in pwRRMS

Sensorimotor variables are often used as proxy measurements for disability severity in pwMS and can provide quantitative information about localized alterations in neurological function (Miehm et al., 2020;Zackowski et al., 2009). Evaluating the relationship between FC using ROI-based analyses and sensorimotor outcomes provides an objective method to probe the correspondence between what a patient experiences and how the SC functions. In fact, ROI-based FC metrics revealed significant associations with sensorimotor impairment. The decline of FC with mobility (Z-scored TUG/T25w as the average mobility Z-scored value between TUG and T25) in the dorsal-dorsal, ipsilateral left, and CRVLD networks suggests greater FC may relate to preserved walking ability and faster mobility times. In the brain of older healthy adults, faster walking speeds (i.e. lower Z-scored TUG/T25w) correlate with increased connectivity between cognitive and motor neural networks and decreased connectivity between cognitive and limbic networks (Poole et al., 2019). We hypothesized increased ventral-ventral FC would relate to lower Z-scored TUG/T25w, though this was the only FC value that did not relate to mobility measures at least at the trend level for adjusted*p*-values. As noted in previous work, our vibration threshold measurements in pwRRMS were not significantly correlated with FC, likely due to our patient population with minimal disability and clinical symptoms, and further work is required using pwRRMS with an EDSS above 4 to better capture FC associations with vibration threshold (A. J. E. Combes et al., 2022).

Similar to reported discrepancies of both increased and decreased correlations among pwMS brain networks, our group level tests of FC differences between HCs and pwRRMS in the SC also did not show a clear trend (Roosendaal et al., 2010). The decrease in ventral-ventral FC between EDSS = 0 and EDSS = 1, followed by slight increase and then steady decrease in ventral-ventral FC, aligns with our observations from ICA and supports the notion of compensatory FC that seems to temporarily protect against increasing disability. In a similar study performed on patients with Parkinson’s disease, the severity according to the Unified Parkinson’s Disease Rating Scale, akin to EDSS used in pwMS, was associated with a decrease in SC FC and provided compelling evidence of altered SC circuitry in neurologic disease (Landelle et al., 2023). Given the established importance of ventral-ventral FC and hypothesized role in motor network synchronization, our findings suggest maintenance of neural integrity may be contributing most significantly to ventral-ventral FC compared with other FC metrics. However, it is important to note the wide distribution of ROI-based FC values across EDSS scores inFigure 3. When looking at ventral-ventral FC values fromTable 2andFigure 2, the FC range extends from 0.331 to 0.816, and though there is greater ventral-ventral FC at an EDSS = 0, it is understandable that the overall effect is blunted by this large distribution. The average C3–C5 lesion burden similarly modeled the slight increase and then steady decrease noted with ascending EDSS scores. As noted in the introduction, correlations between EDSS and lesion measures in pwRRMS are controversial (Stankiewicz et al., 2009), and there were no significant relationships identified between EDSS and lesion burden as part of our work.

Finally, we noted an association between increased FC and females derived from our mobility and vibration models. Females have been shown to have higher FC between SC ventral horns than their male counterparts (Conrad et al., 2018), though in the brain there are reports of both increased and decreased connectivity with sex-related differences. Some reports suggest stronger intra-network FC between resting-state networks and within default mode regions in females, while others suggest male brains are more optimized for intra-hemispheric connection compared with females (Allen et al., 2011;Biswal et al., 2010;Ingalhalikar et al., 2014). Our findings support the notion of sex differences in SC connectivity, with different networks identified compared with the reported sex differences in ventral-ventral FC. It is plausible there are still significant differences in ventral-ventral FC in females that were not captured by our model. Further studies are required before concluding with finality there are established sex differences in SC FC.

The significantly older pwRRMS population was accounted for by including age as a covariate in our statistical analysis; it remains feasible that our population had yet to progress to severe disability levels given the associations between age and attainment of EDSS above 6, though additional work is required to completely parse out age effects from our models (Scalfari et al., 2011).

### 4.2 Preserved regional FC in pwRRMS and HCs

Mean tSNR was consistent with previous groups’ work and across vertebral levels with no significant difference between HCs and pwRRMS, suggesting data acquisition and analysis did not significantly depend on cohort (A. J. E. Combes et al., 2022;Conrad et al., 2018;Eippert et al., 2017). Our findings of significantly increased ventral-ventral connectivity compared with connectivity for all other regions (dorsal-dorsal, ipsilateral left, ipsilateral right, CRVLD, CLVRD) with no significant differences between HCs and pwRRMS in each ROI pair support previous findings (A. J. E. Combes et al., 2022;Conrad et al., 2018;Kong et al., 2014;Weber et al., 2018), albeit this study evaluated these findings in a larger cohort (both pwRRMS and HCs). This supports the notion that highly organized resting-state fluctuations both exist in the SC and are preserved, at least in terms of pure sensory and motor processing, considering ongoing disease processes. The significantly greater dorsal-dorsal, ipsilateral left, and ipsilateral right connectivity compared with CRVLD for HCs and pwRRMS and ipsilateral left and ipsilateral right connectivity compared with CLVRD for pwRRMS suggest contralateral connectivity is the least pronounced out of the six defined networks. This observation is in line with previous reports of diminished contralateral within-slice connectivity for rs-fMRI of the SC (Barry et al., 2018;A. Combes et al., 2023;Eippert et al., 2017), especially with established ventral and dorsal networks relating to motor and sensory function (Kong et al., 2014). It is possible these contralateral connections relate more to commissural circuits and are less robust than the ventral and dorsal networks, with less likelihood of being detected via functional BOLD signal (Kinany et al., 2023). It is also plausible that synchronous activity between contralateral horns at rest may occur transiently, and thus contributed less to the overall BOLD signal captured from our functional imaging (Conrad et al., 2018).

### 4.3 Greater ICA-component connectivity earlier in disease processes

We selected 30 components to optimize anatomically distinct regions from which to draw correlations while maintaining sufficient statistical power by reducing granularity of components (Kinany et al., 2020). We see increased “splitting” of components from bilateral to unilateral locations as the number of components increases, though at a point this splitting diminishes the anatomic relevance of each component; thus, 30 components were selected for the purpose of this analysis. We used a GM-specific mask in which to extract component information, though in future analyses the number of components and the size of the mask could be increased to include full-cord analyses. The ICA identified seven distinct anatomic regions corresponding to known anatomy of the cord as well as the parcellated GM zones shown in the PAM50 atlas, including the intermediate gray zone and central commissure (Diaz & Morales, 2016;Ku & Morrison, 2024). The functional parcellation of the GM suggests an expansion of ROI-related connectivity analyses is necessary to include the intermediate and central regions of the cord, beyond just ventral and dorsal delineation, with findings from other groups (both human and non-human primates), suggesting these components are not noise but rather significant anatomical findings (A. Combes et al., 2023;Sengupta et al., 2021).

ICA-based FC provides an opportunity to assess connectivity patterns in the SC, as well as the relevance of said patterns in disease processes. In looking at the connectivity matrices related to group mean connectivity between HCs and pwRRMS, there were more components related to C3 and C4 than those within the C3–C4 “between-levels” region. The cord segments relating to vertebral levels, such as C3, span greater distances than the segments relating to the cord regions where nerve roots branch off, such as between C3 and C4 (Winegar et al., 2020). Comparing correlations between components for HCs and pwRRMS with an EDSS = 0, all significant correlations showed pwRRMS had increased connectivity compared with HCs. Of these significant components, all but three correlations were inter-level versus intra-level relating to components in the C3, C3–C4, and C4 vertebral levels. These findings suggest the rostro-caudal anatomic organization of the cord is conserved functionally, with more components related across vertebral levels rather than within each level. A similar trend as the group level analysis was noted when observing correlations between individual components, both for inter-level (C3 to C4) and intra-level (C4 to C4) correlations. However, care must be taken not to over-interpret these results as they are intended to highlight the spread and variability in component correlations related to a specific disability group (i.e. EDSS = 0) despite the trend supporting our initial observations. It should also be noted that specific location of lesions was not evaluated for each component, which may alter the connectivity between segments.

To receive a categorization of EDSS = 0, the patient must have no demonstrated disability in any functional system and a normal neurologic examination (Meyer-Moock et al., 2014). This significantly increased FC between components, especially when compared with the EDSS = 1 and EDSS = 2+ groups, suggests pwRRMS demonstrate increased SC FC, perhaps even before the development of clinical symptoms. This trend appears to shift for pwRRMS with an EDSS = 1 and EDSS = 2+, demonstrating decreased connectivity in the SC of pwRRMS compared with HCs. Early disease processes and changes in connectivity may contribute to ensuing dysfunction and disability before faltering (Chiang et al., 2021;Van Schependom et al., 2019); however, we would need longitudinal studies to provide the necessary temporal information to understand whether this change is a compensatory mechanism before becoming exhausted. This observation may be further strengthed with histopathological correlations, particularly to establish what occurs at the cellular and biochemical level in early-stage pwRRMS in the SC (Kreiter et al., 2024).

We cautiously suggest the consideration of early disease, even without disability, as a time when significant functional changes are at play in the cord and may impact disease processes down the line. In line with Combes et al.’s suggestion that increased FC in the early stage of pwRRMS has been observed in the brain and supported by findings in the cord (Basile et al., 2014;Faivre et al., 2016;Strik et al., 2021), we believe our findings support this notion that significant functional changes are occurring before the manifestation of clinical disability, perhaps providing additional rationale for earlier treatment before neurologic examination evolution in pwRRMS.

### 4.4 Limitations and future directions

The limitations in this work are similar to previous work with fMRI in the cord, including our use of a low-pass filter with a high-pass cutoff at 0.10 Hz and the challenges of ensuring accurate alignment between fMRI and template images (Barry et al., 2016;A. J. E. Combes et al., 2022;Powers et al., 2018). In an effort to be conservative, the 0.10 Hz cutoff was selected compared with higher values noted in the brain, and images deemed too poor quality for appropriate fMRI ROI analysis were excluded. Future studies could include a detailed evaluation of the appropriate low- and high-pass filter cutoffs for optimum BOLD signal contrast, or even an investigation into the frequency band most impacted by MS pathology as modeled in primates for SC injury (Sengupta et al., 2021). Additionally, though our selection of images with poor quality was validated across multiple raters, this was still a subjective evaluation that could become more objective using quantifiable metrics. One such measure could include calculation of the fractional amplitude of low frequency fluctuations (fALFF) in each GM horn to determine whether this signal is otherwise consistent among subjects.

It is also interesting to consider our selection of GM horns from which we drew correlations, as WM has recently been shown to demonstrate detectable, biologically meaningful BOLD signal at rest in the primate SC and thus may play a contributing role in the overall SC BOLD signatures we captured from GM (Gore et al., 2019;Sengupta et al., 2024). Furthermore, changes in vascular compliance are known to occur generally with age, and thus vascular effects may be a confound in our interpretation of the BOLD vascular phenomenon though likely mitigated with our relatively young recruited participants. In the brain of pwRRMS with cognitive impairment, local cerebral blood flow abnormalities correlate with altered FC (Jandric et al., 2021). Knowing spinal cord vascular reactivity is spatially dependent (Hemmerling et al., 2024), it would likewise be interesting to consider vascular compliance as a potential covariate.

From a recruitment standpoint, our study included majority female compared with male participants in line with the population demographics of the pwRRMS population. Our inclusion of low-disability participants could be built upon in future studies to include pwRRMS with greater EDSS scores, further describing the trend we see here with increasing and then decreasing FC as disability progresses. Additionally, EDSS is not entirely specific to cervical SC damage, as it corresponds to functional systems outside of the cervical SC [i.e. cerebellum, brain stem, etc.]. We recognize that extrapolation of disability status due to EDSS may be limited by this fact, particularly for our analysis segregated to the cervical SC alone. In addition, downstream exclusion of participants in our ICA produced small sample sizes for each EDSS group (EDSS = 0, EDSS = 1, EDSS = 2+). With these concerns in mind, a more representative standard disability outcome in pwRRMS may be T25w instead of EDSS (Koch et al., 2021), or even introduction of a nine-hole peg test.

For independent component analysis, our decision to use 30 components was based on current literature (A. Combes et al., 2023;Kong et al., 2014;Sengupta et al., 2021) and our trials using 10, 20, 30, 40, and 50 components. Given there is no standard component number for fMRI in the cord, it is plausible this study could be repeated using a different choice of component number, though we feel confident that the use of 30 components sufficiently balanced statistical power with anatomically relevant parcellation. This also aligned with previous work performed at our center identifying seven components per vertebral level in the GM of non-human primates (Sengupta et al., 2021). Selection of too few components may diminish the ability to distinguish inter- or intra-level FC alterations, while too many parcellated components could hamper the ability to infer biologically relevant connectivity. Previous studies employing MELODIC’s automatic dimensionality estimation in the SC determined a range of 20 to 30 components for anatomically appropriate estimation of functional networks (San Emeterio Nateras et al., 2016). While we could hypothetically select for hundreds of subcomponents, our selection of 30 components felt most appropriate given the somewhat limited SC field of view and known motor and sensory network distribution within SC anatomy. Following band-pass filtering, further exclusion of components based on a “power threshold” from neutral activity-related frequencies may more effectively encompass signal-related components and enhance sensitivity for detecting connectivity alterations (Vahdat et al., 2020).

Future analysis should also evaluate lesion size and distribution as factors to consider when understanding through-slice correlations and how component correlations shift depending on lesion load. This would expand upon work performed at 7T demonstrating lesion presence alters ROI FC connectivity depending on location of the lesion within SC (Conrad et al., 2018). Additionally, we did not consider correlations outside of the C3 to C4 vertebral range, including those between non-consecutive vertebral levels (i.e. C3 to C5), though this may have some clinical relevance and could be explored further. In studies employing thermal stimulation and controlled hypercapnia, the BOLD response appears to fluctuate in magnitude and variability depending on vertebral level localization, providing evidence for spatially unique and relevant signal across C3, C4, C5, and beyond (Cohen-Adad et al., 2010;Powers et al., 2018).

## 5 Conclusion

We established, in line with previous studies, the presence of robust BOLD signal fluctuations in the SC at rest in HC and MS cohorts. We investigated SC FC, determining relationships between greater FC and better mobility (faster walking speeds) and diminishing FC with increasing disability, and added to the current body of literature suggesting group-wise differences in connectivity exist in pwRRMS. We also applied ICA, a data-driven fMRI analysis technique, and believe there is greater connectivity in earlier MS disease stages as categorized by EDSS. Additional work is necessary to expand this work to MS populations with greater disability and explore how FC may one day be used as an objective, quantifiable measure of the health and well-being of the SC at any given point in time.

## Data Availability

All data generated or analyzed during the study are described in Methods section of this paper. We welcome collaboration and access to our de-identified imaging and sensorimotor data as well as the code written to analyze it. To abide by local institutional policies, a signed data transfer agreement will be required for access and download.
